# Synthesis and characterizations iron oxide carbon nanotubes nanocomposite by laser ablation for anti-microbial applications

**DOI:** 10.1186/s43141-021-00161-y

**Published:** 2021-05-18

**Authors:** Moatasem Al-Salih, Syakirah Samsudin, Siti Suri Arshad

**Affiliations:** 1 Biology department, Faculty of Science and Mathematics, University Pendidikan Sultan Idris(UPSI), Tanjung Malim, Malaysia; 2 Faculty of Veterinary Medicine, University Putra, Seri Kembangan, Malaysia

**Keywords:** Anti-bacteria, Fe_3_O_4_/CNTs, Nanoparticles, Iron oxide, Multi-walled carbon nanotubes

## Abstract

**Background:**

Environmental contamination by microbes is a major public health concern. A damp environment is one of the potential sources for microbe proliferation. Smart synthesis nanocatalytic coatings on surfaces, food, and material from different pathogen bacteria can inhibit using the Fe_3_O_4_/CNTs as anti-microbial growth can effectively curb this growing threat. In this present work, the anti-microbial efficacy of synthesis of a compound nanoparticle-containing iron oxide-multi-walled carbon nanotube was combined by laser ablation PLAL and explored the anti-bacterial action of colloidal solution of Fe_3_O_4_/CNTs NPs that was evaluated against bacteria which is classified as gram-negative (*Escherichia coli* (*E. coli*), *Klebsiella pneumonia (K. pneumonia*), and also that is identified as gram-positive (*Streptococcus pyogenes* (*S .pyogenes*) and *Staphylococcus aureus* (*S. aureus*) under visible light irradiation.

**Results:**

Doping of a minute fraction of iron(III) salt (0.5 mol%) in a volatile solvent (ethanol) was carried out via the sol-gel technique. Fe_3_O_4_ was further calcined at various temperatures (in the range of 500–700 °C) to evaluate the thermal stability of the Fe_3_O_4_ nanoporous oxidizer nanoparticles. The physicochemical properties of the samples were characterized through X-ray diffraction (XRD), atomic force microscopy (AFM), attenuated total reflectance Fourier transform infrared spectroscopy (ATR-FTIR), and UV–Visible spectroscopy techniques. XRD results revealed that the nanoparticles framework of Fe_3_O_4_ was maintained well up to 650 °C by the Fe dopant. UV–Vis results suggested that absorption property of combination Fe_3_O_4_/CNTs nanopowder by PLAL was enhanced and the band gap is reduced into 2.0 eV.

**Conclusions:**

Density functional theory (DFT) studies emphasize the introduction of Fe+ and Fe2+ ions by replacing other ions in the CNT lattice, therefore creating oxygen vacancies. These further promoted anti-microbial efficiency. A significantly high bacterial inactivation that indicates results was evaluated and that the mean estimations of restraint were determined from triple assessment in every appraisal at 400 ml which represent the best anti-bacterial action against gram-positive and gram-negative microbes.

## Background

In this study, compositing nanoparticles comprising iron oxide inserted with multi-walled carbon nanotubes to create Fe_3_O_4_/CNTs nanocomposite were joined with beat laser expulsion of graphite form and Fe_3_O_4_/CNTs centers around that were inundated in water de-ionization. The beat laser utilized was Nd:YAG laser of 1046 nm frequency at altered laser imperativeness masses that reached 5.22–13.07 J/cm^2^ and a substitute numeral of 100–400 beats [1–4].

The visual characterizations of organized impacts were examined while doping the carbon nanoparticles with Fe_3_O_4_/CNTs cNPs nanocomposite [5, 6] The Ultraviolet-Vis assimilation spectra visions demonstrated a redshift as the doping degrees with iron oxide nanoparticles were expanded. While the photoluminescence of carbon nanoparticles doped Fe_3_O_4_/CNTs, oxide nanoparticles demonstrated a reliable fluorescence outpouring tops in perceptible region at 597 nm upon excitation at a frequency of 250 nm, yet with lower power, as the doping extent extended; this is an aftereffect of the effect of Fe_3_O_4_/CNTs nanoparticles in stifling the carbon nanoparticles fluorescence [7–13]. The anti-bacterial development of fused composite nanoparticles was attempted against four particular microorganism minuscule creatures and two gram-negative (*Escherichia coli* (*E.coli*), *Klebsiella pneumoniae* (*K. pneumoniae*) [8, 9, 14–19].

Likewise, the fundamental technique is fluid substrate methodology in particular gathering of carbon tube nanocomposite masterminded in two laser dynamisms 85 mJ and 250 mJ at that point stimulate it with various Fe_3_O_4_/CNTs, the greatest results were gotten from the 400 μg ml^−1^ of carbon nanotube stimulating with some kind extents of Fe_3_O_4_/CNTs nanocomposite [20, 21]. The composite nanopowder that indicated the finest anti-microbial development in the liquid medium system are attempted ceaselessly procedure the incredible scattering technique and reveal that the best centralization of carbon tubes in concentration at 400 μg ml^−1^ which show the influence in anti-microbial actions that are improved and turned out to be better when it doped with 43% Fe_3_O_4_ [8, 16–20].

## Aims of the study

This study aimed to produce and promote a new model of nanocomposite as anti-microbial and anti-germ contamination that made a new profile of anti-resistance nanometers of microorganism, to open new horizons and renaissance for multiple biological applications through low-cost, non-toxic environmentally friendly nanoprocessors. The motivation behind this examination is the brand new model of nanocomposite by changing titanium dioxide for utilizing and obviously saw just as ease and eco-accommodating uncovering the presence of the “wreck” growth of microbe bacteria and how the mix of nanoparticles increment the counter pathogen action. The points of this work are given by some targets:

1. To prepare colloidal iron oxide carbon nanotube composite by beat laser removal procedure in deionized water and orchestrate in this work.
2. To perform laser ablating in iron oxide particles as target in water to synthesize colloidal iron oxide nanoparticles.
3. To identify surface morphology of the stores and materials that have been concentrated by utilizing nuclear power magnifying instruments (AFM). XRD design examination to show the normal grain size and measurements of nanoparticles.
4. To investigate the anti-bacterial movement of Fe_3_O_4_/CNTs nanotubes against four distinctive microorganism microbes and to investigate the best anti-bacterial action grouping of both alter nanocomposite mixed with Fe_3_O_4_/CNTs.

## Methods

### Conducting the experiment

#### Combination Fe_3_O_4_/CNTs nanopowder

Nano-Fe_3_O_4_/CNTs powder was integrated through a sol-gel strategy utilizing iron(III) salt with an epoxide in a volatile solvent (ethanol) to generate nanoporous oxidizer nanoparticles, isopropanol, and deionized water as beginning materials, prompting the advancement of an airborne based sol−gel strategy for preparing nanoparticles iron-oxide nanoparticles with a high inner surface zone. We have utilized sol−gel responses in the airborne stage utilizing an iron(III) salt with an epoxide in an unstable dissolvable (ethanol) to create nanoporous oxidizer nanoparticles. The porosity of the particles results from the idea of the sol−gel science executed [5–7]. SEM and AFM results show that the vaporized-based science is subjectively like that happening in mass sol−gel union. The oxidizer particles acquired from the air sol−gel test are in the framework demonstrating size range as confirmed by SEM and differential versatility investigation using 3-dimensional AFM [1, 5–7]. Schedules of Fe_3_O_4_/CNTs nanoparticles by Nd: YAG laser (1064 nm, beat span = 9 ns, redundancy recurrence 1 Hz) are working at various energies (80 and 200 mJ) for various removal times (10 min and 20 min). Each example was heavy when removal by an advanced weigher to decide LAL iron in refined deionized dish [1, 3, 13–16].

### XRD

Holland Philips X pert X-ray powder diffraction (XRD) diffractometer using monochromatic high-intensity Cu K, radiation (λ = 0.154056 nm), at a scanning speed of 2"/min from 10" to 60" (2*θ*).

### AFM

It is an apparatus which is used to determine or take a picture of the particles, and it used to determine the particle volume in three-dimension *x*, *y*, and *z*, beside that this apparatus is used to determine the volume distribution of this particles. Angstrom A advanced Inc., USA. Model scanning probe microscope as 3000 A.

### ATR-FTIR, UV–Vis

ATR-FTIR Bruker Model Tensor 27, UV–Visible Spectrophotometer, PG instrument Ltd., double+90Plus.

### Bacterial medium properties

The bacterial medium was made and well-adjusted by correlated with 0.5 Mc-Farland turbidity commonplace (5 × 109 cell in ml^−1^) tubing test. It was additionally weakened to get a last of 5 × 107cell in ml^−1^. All microscopic organism strains were cultured in agar media. The media was immunized by the 0.2 ml/5 ml with either the microbe’s strains, at that point included 0.5 ml of IO-MWCNTs nanoparticles at concentration 50, 100, 200, 400, 600 ml^−1^. The examples were brooded at 37 °C. The bacterial development was estimated by an optical thickness that assimilates firmly at 532 nm frequency by spectrophotometer.

## Results

**Fig. 1:**
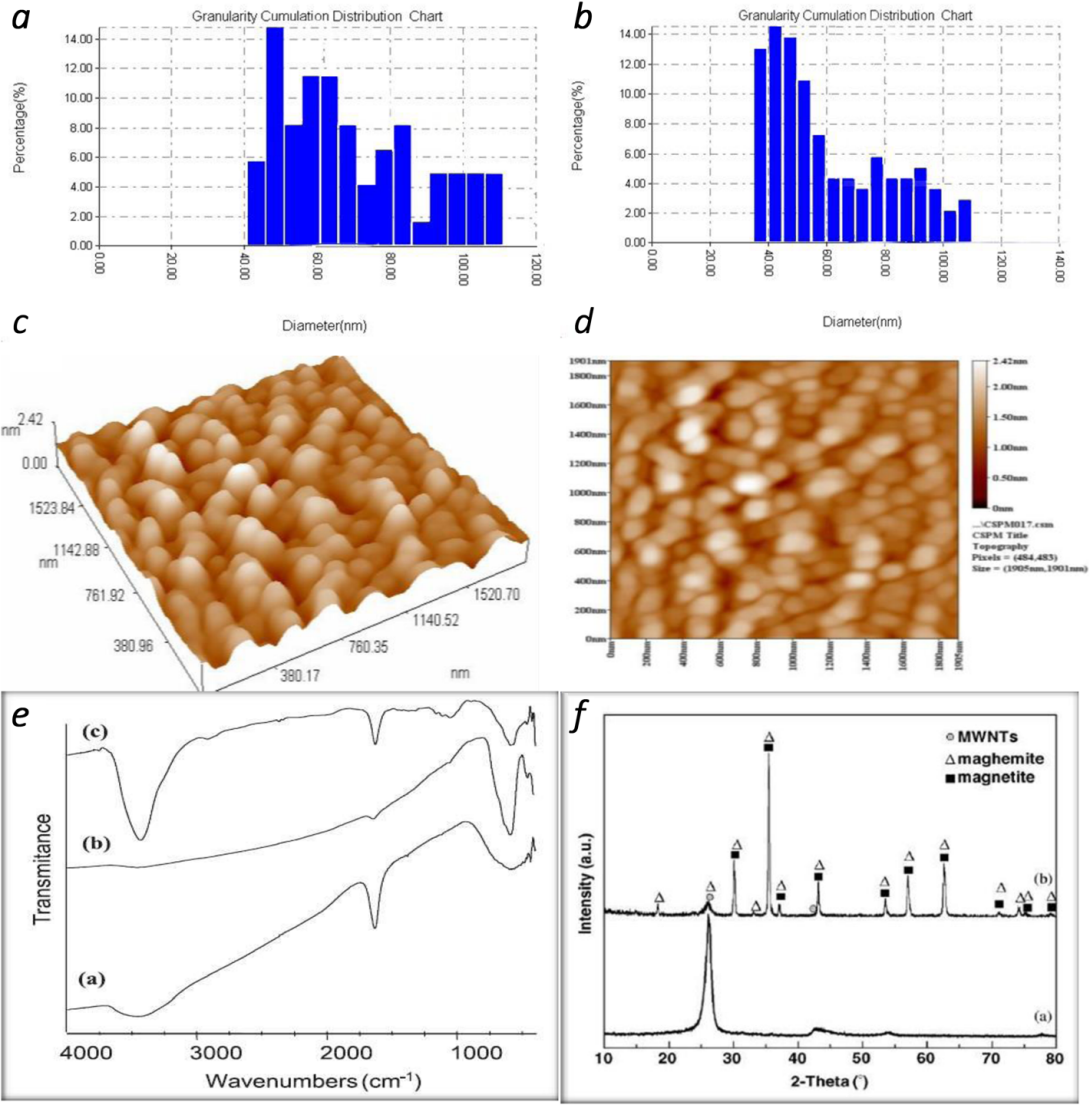
AFM of Fe_3_O_4_/CNTs framework demonstrated **a**, **b** size distribution for catalyst Fe_3_O_4_/CNTs particle by AFM_._ **c** Prepared particles sketched on *X*–*Y* axis catalyst, while the **d** shows prepared particles sketched on *X*–*Y*–*Z* axis for catalyst. **e** FTIR spectra of **a** CNTs, **b** Fe_3_O_4_, and **c** Fe_3_O_4_/CNTs composite, **f** XRD pattern of **a** Fe_3_O_4_/CNTs and **b** decorated Fe_3_O_4_/CNTs (maghemite and magnetite)

XRD charts (Fig. 1d) show that the four integrated examples have the most noteworthy diffraction top in the glass-like plane (A) (2*θ* = 29.9202) and that the other diffraction tops agree with the translucent periods of (B) (25.9348), (C) (25,3439) and the littlest vertex of (E) (33,9715). These outcomes have indicated that we can plainly observe that the translucent period of each example is masculine in anatase structure. This outcome corresponds with [1, 2]. AFM spectra showed (Fig. 1a, b) the moment size conveyance between 60 and 135 nm for Fe_3_O_4_/CNTs and the moment size dispersion between 50 and 150 nm, for Fe_3_O_4_/CNTs doped with water orchestrated at 873 k. The results show that the Fe_3_O_4_/CNTs doped with water has the biggest surface zone, trailed by Fe_3_O_4_/CNTs which has a littler surface territory contrasted with the reduction in molecule size D Avg = 91.24 nm and the dimer (Fig. 1c) high *Z* = 0.30 nm between the particles is 0.30 nm (1) discovered goes with [5–7]. The outcomes likewise indicated that an exceptionally noteworthy distinction between the gatherings considered (*P* < 0.000) demonstrated that a high focus in ladies was a lot higher than in the male, which affirms that the presence of the impact is commonly an expansion in the fixation did by introduction to nanoparticles. The outcome likewise demonstrated that distinctions were found between the treatment gathering and the benchmark group; so, this finding concur with [7] and [9], respectively.

### Morphological properties

**Fig. 2:**
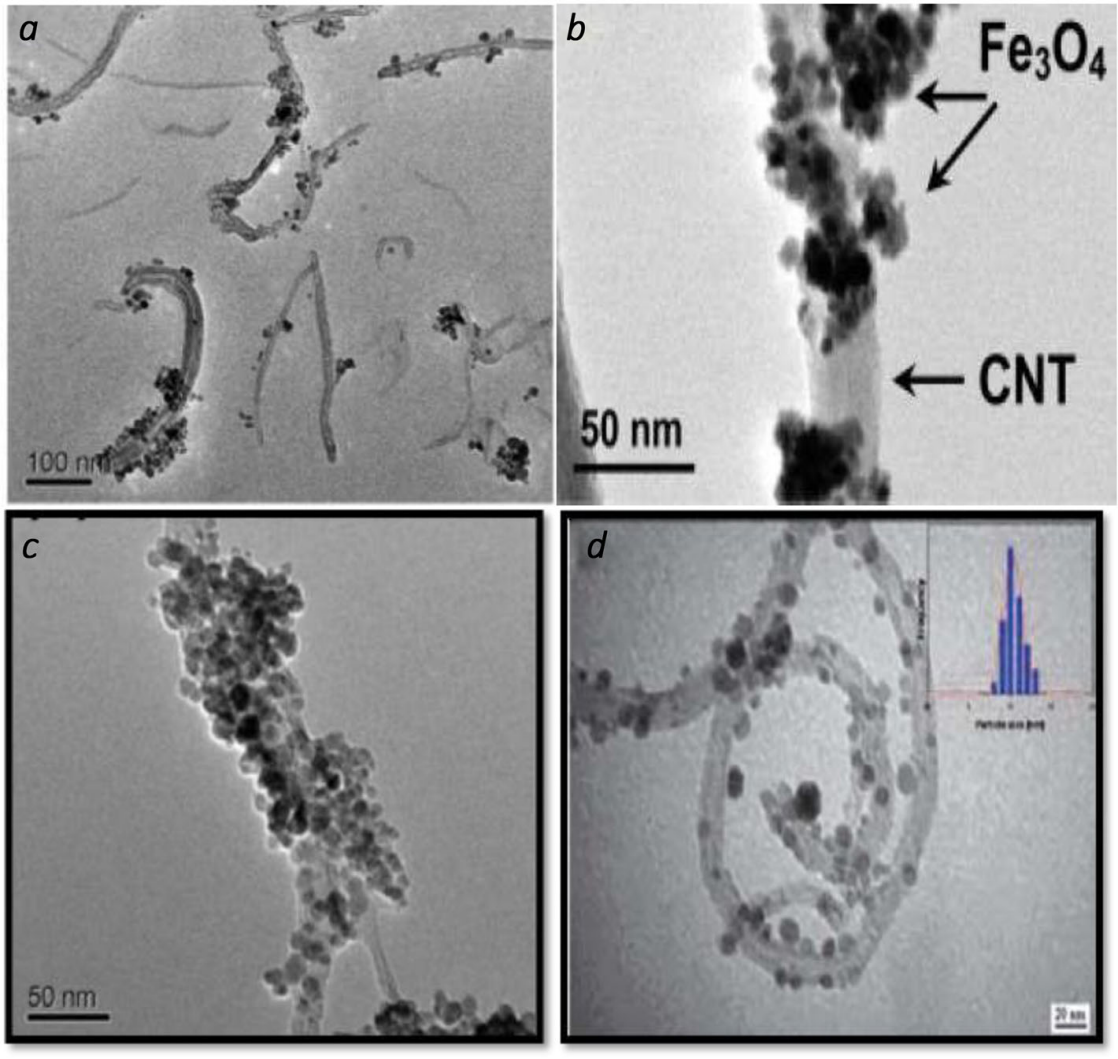
TEM images of the (**a**), (**b**), (**c**) magnetic nanotube Fe_3_O_4_ / MWCNTs at two magnification, and (**d**) TEM images of maghemite γ–-Fe_2_O_3_

Figure 2 shows the TEM images of the prepared magnetic nanotube Fe_3_O_4_/MWCNTs samples containing elemental iron. The Fe3O4 nanoparticles appear as spherical nanocrystals that spread on the sidewalls of MWNTs; most tubes were loaded with iron oxide nanoparticles and this can clearly be observed at higher magnification as shown in Fig. 2b that some nanoparticles assembled into nanoclusters. For some individual tubes, the nanoclusters ordered with the iron oxide nanoparticles can completely enwrap the tubes, producing core shell magnetic nanostructures (Fig. 2c). The iron oxide nanoparticles attached strongly and perfectly on the nanotube surface and they look like nodes rising from the nanotube. Figure 2d shows TEM images of MWCNTs/Fe_2_O_3_ that Fe_2_O_3_ was attached to the walls of the MWCNTs representing the high crystallinity of the maghemite nanoparticles [8, 9, 14–19].

## Discussion

The optical properties of prepared collide were investigated before and after doping the carbon nanoparticles with iron oxide nanoparticles. The UV–Vis absorption spectra exhibited a redshift as the doping ratios with iron oxide nanoparticles were increased, while the photoluminescence of carbon nanoparticles doped with iron oxide nanoparticles exhibited a constant fluorescence emission peaks in the visible region at 597 nm upon excitation at a wavelength of 250 nm, but with lower intensity, as the doping ratio increased; this is because of the effect of iron oxide nanoparticles in quenching the carbon nanoparticles fluorescence this results goes with [1, 3, 13–15].

The structural study of composite carbon nanoparticles doped with different iron oxide nanoparticles has been confirmed using Fourier transform infrared spectrum (FTIR) and X-ray diffraction investigation (XRD). From the FTIR results, it has been successfully found that the C–H C=C, C≡C, C=O, C=O=C, different iron oxide bonds, and CNT-IO are confirmed by pulsed laser ablation in the liquid process. XRD patterns that took over a scanning interval from 20° to 80° proved the presence of (02) and (100) planes which belong to graphene layers of multi-walled carbon nanotubes and the honeycomb lattice of the single graphene sheet, also the existent for different planes of iron oxide nanoparticles according to [1, 3, 8, 9].

Morphological properties of composite carbon nanoparticles doped with iron oxide nanoparticles were investigated by transmission electron microscopy(TEM) and energy dispersive spectrum (EDS) are measured; the TEM measurement showed an individual and two straight long multi-walled carbon nanotubes MWCNT with a hollow core adhered. While for composite nanoparticles, the TEM study reveals that there are two types of composite carbon and iron oxides nanoparticles are present; the first type is the iron oxide nanoparticles that attached to the walls of CNT, and the second rare type is carbon-coated iron oxide nanoparticles. The EDS measurements reveal the content of carbon, iron, and oxygen. Readiness strategy utilized prompted getting Fe_3_O_4_/CNTs nanoparticles measurements adjusting the band hole and prompted getting a littler band hole (2.0 eV) Fe_3_O_4_/CNTs. XRD, AFM gem size, surface morphology, and molecule size and surface geology properties to all examples demonstrated the effective sights of the readied mixes these outcome goes with 14–17, 20–21].

**Fig. 3:**
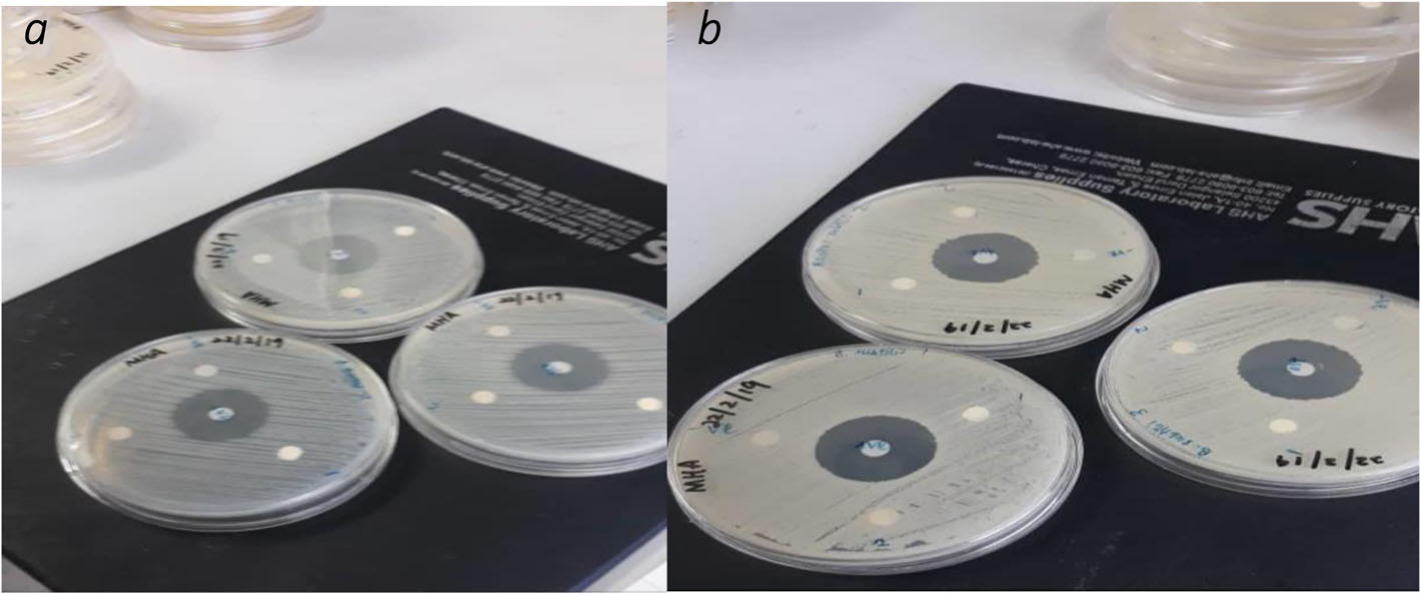
This figure showed the difference between effective nanoparticles in bacteria growth in media these particles which experimenting configuration in Laser ablation in liquefied setups where a focused beam irradiated; bacterial target placed in pure melted a colloidal solution of nanoparticles. **a**) refer to replicate in three madia in *E. coli*. **b**) *K. pneumonia* *n* = 3

The anti-bacterial movement of combined merged nanocomposite was tried against four diverse microbe microorganisms: two gram-negative (*Escherichia coli (E. coli*), *Klebsiella pneumonia* (*K. pneumoniae*)) and two kinds of gram-positive (*Streptococcus pyogenes*) and Staphylococcus aureus) with dual strategies; principal strategy is fluid culture cycle that is diverse grouping of nanocomposite arranged in dualistic laser powers that is 80 mJ and 200 mJ at that point fixing it with various iron oxide nanopowders. The greatest outcomes were acquired from the 400 μg ml^−1^ of Fe_3_O_4_/CNTs nanocomposite drugged with various proportions of nanoparticles. The sterile activity of combination of carbon tube and iron oxide nanocomposite was performed as assay in contradiction of four categories of pathogens; two types of them were the distinction of being gram-negative of bacterial wall as *Escherichia coli*, *Klebsiella pneumoniae*, and the other two kinds of them which are distinct being gram-positive wall like *Streptococcus pyogens* and *Staphylococcus aureus* by well-dispersion techniques. The hatched microscopic organism’s media prior to adding the composite of particles appeared in Figs. 3, 5, and 6. The suspension fixations utilized were 400 μg ml^−1^ of just carbon nanotubes utilized as control, and afterward doped with iron oxide NPs of three distinct focuses (100, 250, 300 μg ml^−1^), which spoke to by doping proportions (20, 38, 43%) separately, gotten by laser removal of carbon and iron focuses in deionized water at a laser energy thickness of 5.2 J/cm^2^. Composite nanoparticles, which have demonstrated the best anti-bacterial activity in a fluid medium technique, are continually tried in a well-dissipating technique procedure and find that the best combinations of 400 μg/ml carbon nanotubes, which show the best anti-bacterial activity, are improved and end up being better when doped with 43% nanoparticles that indicate the outcome correspondence with [14–19].

**Fig. 4:**
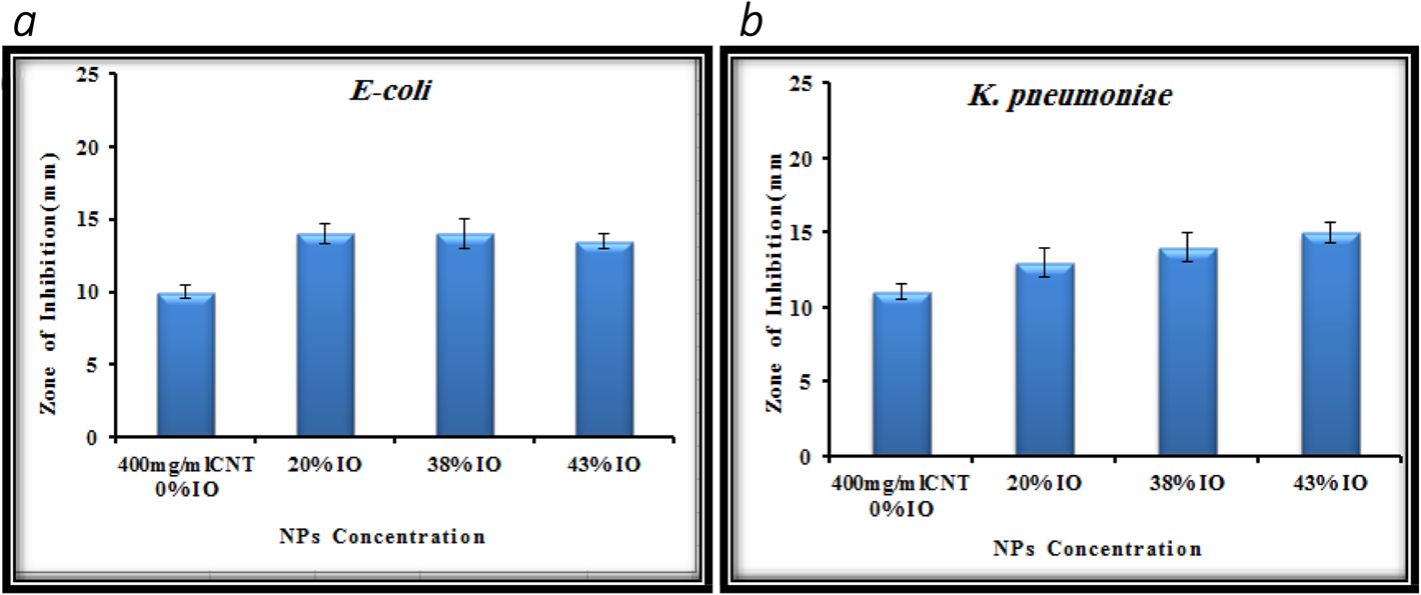
Histograms of Anti-microbial growth activity stimulated by IO-MWCNTs. Composite against two types of bacteria gram negative: (**a**) *E. coli*, (**b**) *K. pneumonia*

**Fig. 5:**
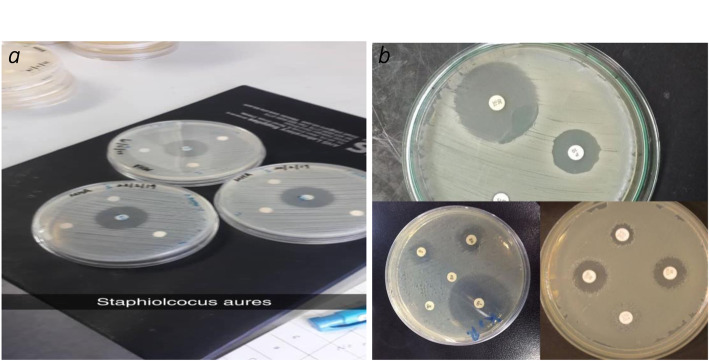
demonstrate the histograms and inhibition zone (IZ) image respectively of the anti-bacterial growth in media (**a**) against gram-positive *Streptococcus pyogens* (*S. pyogenes*) replicate three media *n* = 3. (**b**) in *Staphylococcus aureus* (*S. aureus*) pathogens, which induced by carbon nanotubes at 400 μg mL^−1^ concentration and composite carbon nanotubes doped with Iron Oxide NPs at three different concentrations ratios (20, 38, 42%) in three media

As shown in Fig. 4, determining the inhibiting zone IZ region by capacity of that nobbling the iron oxide-MWCNTs nanocomposite container improved the anti-growth of bacteria activity interaction with pathogen culture distinguishing gram-negative, and this activity increased clearly with the increase of the IO NPs concentrations. Figure 3 exhibits the histograms of the anti-bacterial activity against two gram-negative *E. coli* and *K. pneumoniae* pathogens which were induced by carbon nanotubes at 400 μg ml^−1^ concentration and composite multi-walled carbon nanotubes doped with iron oxide NPs at three different concentrations ratios (20, 38, 42%). Figure 5 shows the inhibition zone (IZ) image of carbon nanotubes of 400 μg ml^−1^ which represented as control without adding iron oxide nanoparticles, and composite carbon and iron oxide NPs in three concentrations that maintained above according to [8, 9, 20, 21].

**Fig. 6:**
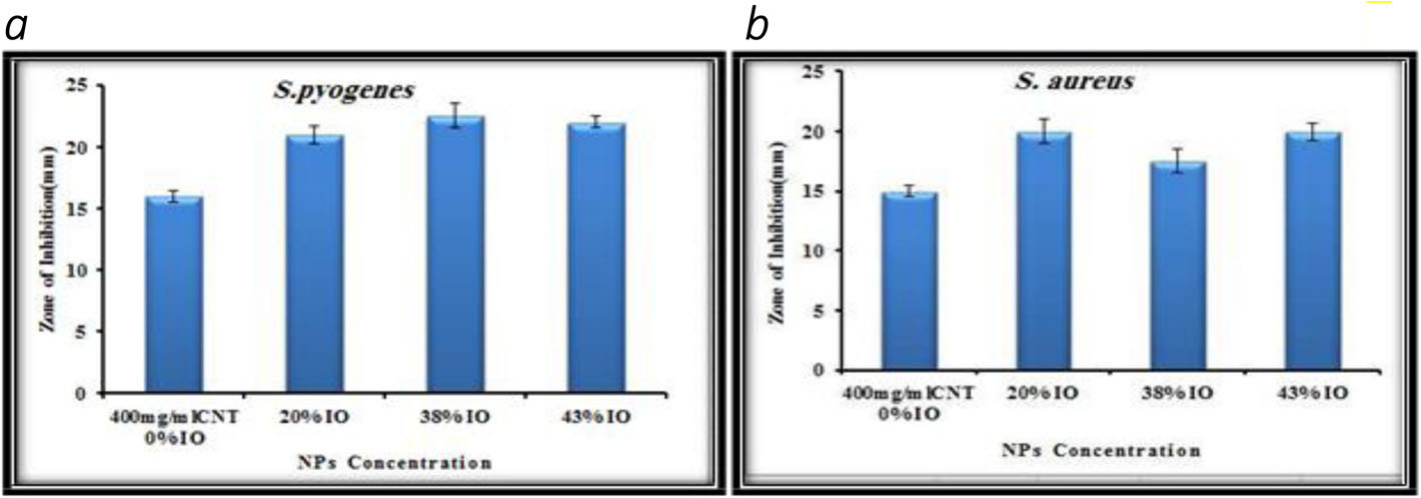
Images and histograms of Anti-bacterial growth reduced by IO-MWCNTs and composite that’s dubbing IO-MWCNTs NPs against two gram-positive (**a**) *S. pyogenes* and (**b**) *Staphylococcus aureus* (*S. aureus*)

Figures 5 and 6 demonstrate the histograms and inhibition zone (IZ) image, respectively, of the anti-bacterial growth in media against gram-positive *Streptococcus pyogens* (*S. pyogenes*) and *Staphylococcus aureus* (*S. aureus*) pathogens which were induced by carbon nanotubes at 400 μg ml^−1^ concentration and composite carbon nanotubes doped with iron oxide NPs at three different concentrations ratios (20, 38, 42%) that goes with [4, 8, 9, 14, 15].

At last, utilizing the Fe_3_O_4_/CNTs as anti-microbial was tested against four different pathogen bacteria two gram-negative (*Escherichia coli* (*E. coli*), *Klebsiella pneumoniae* (*K. pneumoniae*)), and two gram-positive (*Streptococcus pyogenes* (*S. pyogenes*) and *Staphylococcus aureus* (*S. aureus*)) by two methods: the first method is a liquid medium method in which different concentrations of multi-walled carbon nanoparticles were prepared in two laser energies (80 mJ and 200 mJ) then doping them with different iron oxide nanoparticles; the best results were obtained from the 400 μg ml^−1^ multi-walled wall carbon nanoparticles doped with different ratios of iron oxide nanoparticles. The composite nanoparticles that exhibited the best anti-bacterial activity in the liquid medium method are tested by the second method, the good diffusion method, and reveals that the best concentration of multi-walled carbon nanotubes (400 μg ml^−1^) that exhibit the best anti-oval activity are enhanced and become better when it doped with 43% iron oxide nanoparticles.

## Conclusions

As a result of this work, the end could be summed up as follows:

1. For optical properties, the absorbance of both carbon nanotubes and iron.
2. Oxide nanoparticles were expanded as laser energy and removal time were expanded as well, while the photoluminescence (PL) of carbon nanotubes diminished with expanding the doping proportions of iron oxide NPs.
3. Carbon NPs were incorporated in two structures (nanotubes and graphene sheet), and the iron oxide nanoparticles with round shapes were totaled around the carbon nanotubes; this accumulation expanded as laser removal time emerges. The second type of orchestrated composite NPs was seen as iron oxide NPs encompassed with graphene sheet.
4. The best anti-bacterial action against gram-positive and gram-negative microbes was for the fixations 400 μg/ml doped with various proportions of iron oxide nanoparticles more than for 200 μg/ml doped additionally with various iron oxide proportions because of the impact of arranged nanoparticles size.
5. The anti-bacterial movement for the composite carbon nanotubes doped with iron oxide nanoparticles was higher against gram-positive *Streptococcus pyogenes* (*S. pyogenes*) and *Staphylococcus aureus* (*S. aureus*) than for gram-negative microbes *E. coli* and *K. pneumonia*.

## Data Availability

We have taken due care that the scientific knowledge, historical data, evidential artifacts, figures, and all other statements contained in the article conform to true facts and authentic formulae and will not, if followed precisely, be detrimental to the user. We permit the adaptation, preparation of derivative works, oral presentation, or distribution, along with the commercial application of the work. Copyright is given at the Org level and so Journal of Genetic Engineering and Biotechnology can determine the journal in which the paper is to be published or change the journal at any point in time in which the paper is to be published.

## Abbreviations

NPs: Nanoparticles
Fe_3_O_4_/CNTs: Iron oxide/Carbon Nanotube
MWCNTs: Multi-walled carbon nanotubes
E. coli: *Escherichia coli*
K. pneumonia: *Klebsiella pneumoniae*
S. pyogenes: *Streptococcus pyogenes*
S. aureus: *Staphylococcus aureus*
ATR-FTIR: Attenuated total reflectance Fourier-transformed infrared spectroscopy
XRD: X-ray diffraction
TEM: Transmission electron microscopy
AFM: Atomic force microscopy
